# Gadobenate dimeglumine (MultiHance) or gadoterat emeglumine (Dotarem) for brain tumour imaging? An intra-individual comparison

**DOI:** 10.1186/1470-7330-15-S1-P15

**Published:** 2015-10-02

**Authors:** M Vaneckova, M Herman, MP Smith, M Mechl, KR Maravilla, C Colosimo, A Bonafé, S Lui, MA Kirchin, G Pirovano

**Affiliations:** 1 Bracco Imaging SpA, Milan, Italy

## Purpose

To intra-individually compare 0.1 and 0.05 mmol/kg gadobenate (MultiHance) with 0.1 mmol/kg gadoterate (Dotarem) for contrast-enhanced MRI of brain tumours.

## Method and materials

One hundred seventy-seven adult patients with suspected or known brain tumours were randomised to one of two study arms to undergo two identical exams at 1.5T, one with gadobenate at 0.1 (Arm 1; 70 patients) or 0.05 (Arm 2; 107 patients) mmol/kg bodyweight, and the other with gadoterate at 0.1 mmol/kg bodyweight. The agents were injected in randomised order separated by 2–14 days. Imaging sequences (T1SE and T1GRE) and acquisition timing were identical for the two exams. Three blinded readers evaluated images qualitatively for diagnostic information (lesion extent, delineation, morphology, enhancement, global preference) and quantitatively for % lesion enhancement and lesion-to-background ratio (LBR). Safety assessments were performed.

## Results

In Arm 1, readers 1, 2 and 3 demonstrated highly significant global preference for gadobenate (31 vs. 1, 51 vs. 2, 43 vs. 2 patients, respectively; p<0.0001, all readers), all other qualitative endpoints (p≤0.0023; all readers) and for % enhancement (p≤0.0006) and LBR (p<0.0001). Significant (p=0.023) differences between 0.05 mmol/kg gadobenate and 0.1 mmol/kg gadoterate (in favour of gadobenate) were noted only by reader 2 for % enhancement. Study agent related adverse events were reported by 2/169 (1.2%) patients after gadobenate and by 5/175 (2.9%) patients after gadoterate.

## Conclusion

Significantly superior morphologic information and contrast enhancement are demonstrated on brain MRI with 0.1 mmol/kg gadobenate compared to 0.1 mmol/kg gadoterate. No meaningful differences were recorded between 0.05 mmol/kg gadobenate and 0.1 mmol/kg gadoterate.

